# Heightened Negative Affects Associated With Neurotic Personality in Behavioral Addiction

**DOI:** 10.3389/fpsyt.2020.561713

**Published:** 2020-09-03

**Authors:** Yui Asaoka, Moojun Won, Tomonari Morita, Emi Ishikawa, Yukiori Goto

**Affiliations:** ^1^ Primate Research Institute, Kyoto University, Inuyama, Japan; ^2^ Kyowa Hospital, Obu, Japan

**Keywords:** anxiety, cortisol, depression, impulse control disorder, kleptomania, paraphilia, personality, stress

## Abstract

Although studies have demonstrated that negative affects are critical attributes of drug addiction, this has remained less clear in behavioral addiction. In this preliminary study with a relatively small number of samples, we investigated negative affects in patients diagnosed with behavioral addiction, particularly paraphilia and kleptomania. Negative affects were examined using self-rating questionnaire and further evaluated by objective assessments in behavioral addicts and normal subjects. Explicit, self-referential negative affects, such as anxiety, stress, and depression, were higher in behavioral addicts than control subjects. Such self-referential negative affects were, although not entirely, consistent with objective evaluations by others and blood stress hormone concentrations. Further investigation of personality traits in behavioral addicts unveiled that heightened negative affects were associated with stronger neurotic personality in behavioral addicts than normal subjects. These results suggest that behavioral addiction, such as paraphilia and kleptomania, may be characterized by heightened negative affects attributable to stronger neurotic personality.

## Introduction

Behavioral addiction (BA) is a psychiatric condition with an intense desire to repeat an action that is rewarding or alleviating distress, despite negative consequences (1–4). BA is hence characterized by impulsive initiation of an action and subsequent development of compulsive seeking of the action (1–4). Owing to such characteristics, impulse control disorders, such as kleptomania (KM) and compulsive sexual behavior, are thought to meet the criteria of BA, and thereby often considered as this category of disorders (5–8). Compulsive sexual behavior is excessive or uncontrolled sexual behaviors or thoughts that are either nonparaphilic or paraphilic (PP), whereas KM is characterized by repetitive, uncontrollable stealing of items for unintended personal use. Although the 5th version of Diagnostics and Statistical Manual of Mental Disorders (DSM-5) has included pathological gambling in the “Substance Related and Addictive Disorder” as a prototypical BA, KM and PP have remained within the category of disruptive, impulse control, and conduct disorders (9, 10), primarily due to insufficient studies on KM and PP. We have recently reported that patients diagnosed with KM and PP exhibit impaired probability judgements and risk-taking associated with compromised right prefrontal cortical (PFC) activity (11). Similar alterations of PFC activity in probabilistic decision-making have been reported in patients with pathological gambling (12) and drug addiction (13), suggesting that the mutual neural deficit may be involved in both impulse control disorders such as KM and PP and addiction. Collectively, however, further investigations are required to establish the concept and definition of BA.

Affective disorders are one of predominant components in a range of psychiatric disorders, and negative affects, such as anxiety, depression, and stress, are subjected for treatment interventions (14, 15). Accumulating evidence suggests that alterations of negative affects play important roles in addiction (16, 17). In drug addiction, depressive and anxiety disorders are frequently co-morbid (18–20). On the other hand, withdrawal symptoms also facilitate negative emotional arousals, such as anxiety and depression, in drug addicts (19). Stress plays critical roles in drug addiction. For instance, stress could be a risk factor for not only urges of symptoms but also subsequent relapse after treatments (21, 22). Impulse behavior has been shown to develop in response to stress due to deficits in regulatory control over emotional and motor-related behaviors (22, 23). Drug addicts with co-morbidity of posttraumatic stress disorder exhibit stronger form of impulsive behavior, including aggression (24). Accumulating evidence suggests that BA is similarly associated with negative affects, such as depression and anxiety, independent of symptom types (25–27).

Affects have been demonstrated to have tight relationships with personality (28). The five-factor model of personality, or the Big Five personality traits, explains that the personality can primarily be divided into five basic dimensions of traits, i.e., extraversion, conscientiousness, agreeableness, neuroticism, and openness (29). Subjects with a strong neurotic personality trait exhibit the higher level of negative affects, such as anxiety, stress, and depression, whereas a lower level of neuroticism is associated with better emotional regulations (30, 31). Accordingly, neuroticism has quite often been associated with higher susceptibility of psychiatric disorders (32, 33). Although inconsistent, studies have also shown that individuals with BA exhibit personality traits associated with high anxiety, aggression, and neuroticism (34, 35). However, affective disorders in BA has remained less explored and less understood than drug addiction to date.

Considering these previous studies demonstrating higher negative affects in addiction in both drug addiction and BA, negative affects may be the important attribute of addiction in general. In this preliminary study with a relatively small sample size, we investigated whether negative affects were also higher in patients diagnosed with BA, which primarily consisted of KM and PP, than healthy people, and whether associations between negative affects and personality were observed in BA to provide a better appraisal of this psychiatric condition.

## Methods

### Subjects

This study was conducted in accordance with the Declaration of Helsinki and the Ethical Guidelines for Medical and Health Research Involving Human Subjects by Japanese Ministry of Health, Labour and Welfare. All experimental procedures were approved by the Human Research Ethics Committee of Kyoto University Primate Research Institute, and the Ethics Committee of Kyowa Hospital. Written informed consents were obtained from all participants in advance of experiments.

Sixteen hospitalized patients diagnosed with BA (20–72 years old; 56% males and 44% females) who were divided into gambling (n = 1), kleptomania (KM; n = 10) and paraphilia (PP; n = 5) were recruited. As a control (CT) group, 31 healthy adult subjects (18–58 years old; 41% males and 59% females) without a history of psychiatric disorder and smoking in the past 6 months were recruited. Subjects whose full-scale intelligence quotient (FIQ) was estimated below 60 and/or who were unable to understand instructions for the tests were excluded from the study.

### Questionnaires

First, we conducted an explicit, self-rating questionnaire survey of negative affects in hospitalized BA patients. Three questionnaire surveys were conducted, of which two were aimed to assess negative affects, and one was for personality traits, respectively, of participants. All questionnaires were in the Likert format and were translated in Japanese from the original English versions.

Negative affects were evaluated using the 21-item version of depression anxiety stress scale (DASS21) and Hamilton anxiety rating scale (HAM-A). DASS21 is a well-established, self-report questionnaire designed to measure symptoms of depression, anxiety, and stress, in both clinical and non-clinical samples of adults (36). HAM-A is a widely used 14-item clinician-administered rating questionnaire to measure severity of anxiety symptoms in adults (37). In this study, the medical attendant who was taking care of recruited BA patients scored this questionnaire for BA patients and a part of CT subjects, who were co-workers of the medical attendant (n = 24).

To provide an insight on why negative affects were higher in BA patients, we investigated whether trait personality of subjects might be associated with negative affects using the Japanese version of ten item personality inventory (TIPI-J). TIPI-J is a brief questionnaire measuring Big Five personality traits; extraversion, agreeableness, conscientiousness, neuroticism (emotional instability), and openness (38, 39). The questionnaire comprises 10 items, with 2 items each for assessing one personality trait (one item for the positive pole and the other for the negative pole). Participants self-rated how each trait applied to themselves using a seven-point Likert scale.

Since affects are often processed unconsciously (40, 41), it is possible that explicit, self-referential assessments of negative affects with the questionnaire may not accurately reflect the states of negative affects in participants. Thus, we further evaluated negative effects of BA patients and CT subjects with objective measurements. For BA patients, this questionnaire was also rated by the medical attendant to evaluate a consistency between self- and objective-rating.

In this study, the Japanese version of questionnaires were used, all of which have been validated with large sample sizes of Japanese population. DASS-21 available at http://www2.psy.unsw.edu.au/dass/Japanese/Japanese.htm was translated by John Naaykens, the certified school psychologist, and tested at multiple higher educational institutes in Japan. The Japanese version of HAM-A is commercially available, and has been widely utilized in medical institutes, along with one of recent validation studies by Otsubo and colleagues (42). TIPI-J has been developed and validated in the study by Oshio and colleagues (38). These have now been described in the revised manuscript.

### Stress Hormone Assay

Given that our samples were relatively small, we further evaluated such self-reported data of negative affects by conducting objective assessments, in which rating of negative affects was conducted by others and blood stress hormone assay with enzyme-linked immunosorbent assay (ELISA) to examine consistency between self-referential and objective rating of negative affects.

Cortisol concentrations in whole blood samples obtained from participants were measured with ELISA to implicitly estimate stress level. Blood samples were collected from BA patients around the noon (11:00–11:30) and from CT subjects in the afternoon (between 15:00 and 17:00) 1 day prior or on the day of other tests and were stored in the freezer at −30°C until the days of processing for ELISA. Sample processing was conducted using a commercially available human ELISA cortisol assay kit from Arbor Assays (catalog no. K003-H1/H5) according to the manual. After processing, ELISA plates were read using iMark microplate Reader (Bio-rad, Hercules, CA).

### Data Analysis

Investigators who were not blinded to the experimental conditions collected the data for statistical analyses. All statistical analyses were conducted using Statistica software (StatSoft, Tulsa, OK, USA). A probability value of p < 0.05 was considered as statistical significance. Statistical analysis of the data was conducted using parametric tests. When ANOVA was used for analysis, Tukey test was conducted for post-hoc pair wise comparison. Linear correlation and multiple regression analyses were employed to examine correlations between different assessments.

## Results

### Self-Referential Negative Affects

CT subjects (n = 31) and hospitalized BA patients (n = 16) who were diagnosed with the symptoms of pathological gambling (n = 1), KM (n = 10) or PP (n = 5) were recruited.

**Figure 1:**
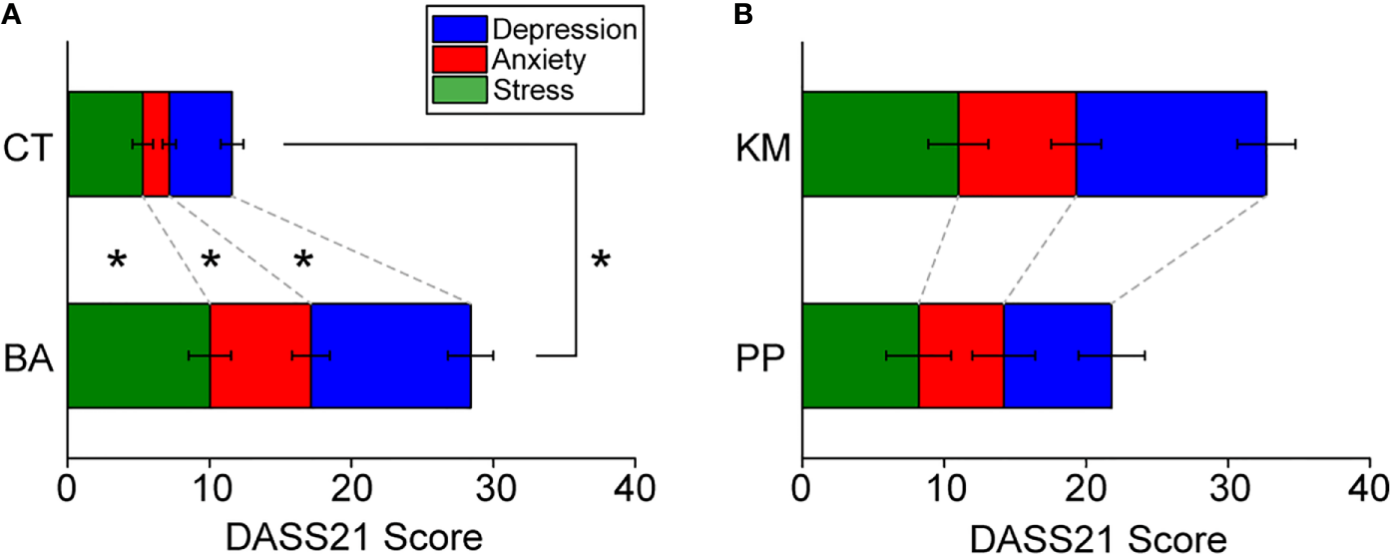
Self-assessment of negative affects with 21-item version of depression anxiety stress scale (DASS21). **(A)** A stacked bar graph showing scores in stress, anxiety, and depression scales of DASS21, respectively, in behavioral addiction (BA) patients and control (CT) subjects. Error bars indicate s.e.m. *p < 0.05. **(B)** A bar graph similar to **(A)** but showing those in kleptomania (KM) and paraphilia (PP) patients.

Explicit, self-referential negative affects, such as anxiety, stress, and depression, were assessed with DASS21 (36). Two-way ANOVA with post-hoc pair-wise comparison revealed that all of stress (F_1,135_ = 46.8, p < 0.001 in Group; F_2,135_ = 6.89, p = 0.001 in Facet; F_2,135_ = 0.572, p = 0.568 in interaction; post-hoc Tukey test, p = 0.010 in BA vs. CT; **Figure 1A** and **Table 1**), anxiety (p = 0.003 in BA vs. CT; **Figure 1A** and **Table 1**), and depression (p < 0.001 in BA vs. CT; **Figure 1A** and **Table 1**) scales were higher in BA than CT subjects. When BA patients were separately analyzed into KM and PP patients, although an overall trend of difference was observed between KK and PP patients, there was no statistically significant difference in any scales (F_1,39_ = 3.76, p = 0.060 in group; F_2,39_ = 1.14, p = 0.330 in facet; F_2,39_ = 0.340, p = 0.714 in interaction; **Figure 1B**).

**Table 1:** A summary of results for DASS21, HAM-A, cortisol assay, and TIPI-J in BA patients and CT subjects.

		BA	CT
**DASS21**	*Stress*	10.0 ± 1.49*	5.23 ± 0.73
	*Anxiety*	7.13 ± 1.34*	1.87 ± 0.47
	*Depression*	11.3 ± 1.59*	4.42 ± 0.81
	*Total*	28.4 ± 3.89*	11.5 ± 1.69
			
**HAM-A**	**	7.69 ± 1.17*	1.83 ± 0.47
			
**Cortisol (pg/mL)**		408 ± 52.2*	278 ± 25.9
			
**TIPI-J (self-rating)**	*Extraversion*	7.31 ± 0.60	9.32 ± 0.45
	*Agreeableness*	9.19 ± 0.79	10.8 ± 0.36
	*Conscientiousness*	8.19 ± 0.60	6.94 ± 0.46
	*Neuroticism*	9.88 ± 0.80	7.45 ± 0.38
	*Openness*	8.44 ± 0.83	8.29 ± 0.53
			
**TIPI-J (other-rating)**	*Extraversion*	8.06 ± 0.67	—
	*Agreeableness*	6.06 ± 0.38	—
	*Conscientiousness*	7.63 ± 0.88	—
	*Neuroticism*	10.1 ± 0.71	—
	*Openness*	5.56 ± 0.36	—

*Statistically significant difference compared to CT (p < 0.05).

DASS21, 21-item version of Depression Anxiety Stress Scale; HAM-A, Hamilton Anxiety Rating Scale; TIPI-J, Japanese version of Ten Item Personality Inventory; BA, Behavioral addiction; CT, Control; —, Not tested.

These results suggest that BA patients were recognizing higher negative affects than CT subjects.

### Objective Rating of Negative Affects and Correlation With Self-Assessments

**Figure 2:**
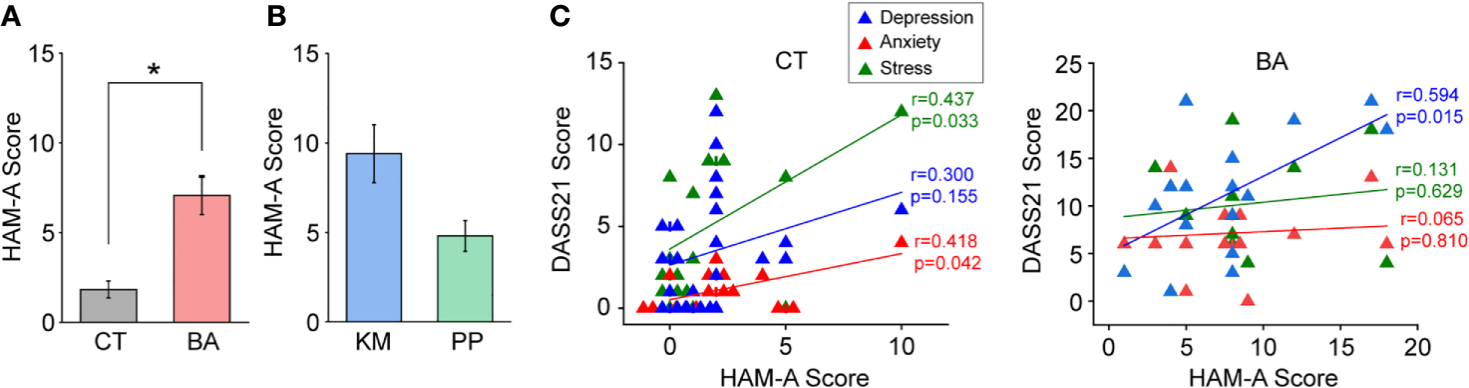
Objective assessment of anxiety with Hamilton anxiety rating scale (HAM-A) and correlations with self-assessment of negative effects. **(A)** A bar graph showing HAM-A scores in between behavioral addiction (BA) patients and control (CT) subjects. Error bars indicate s.e.m. *p < 0.05. **(B)** A graph similar to (A) but showing those in kleptomania (KM) and paraphilia (PP) patients. **(C)** Graphs showing correlations between HAM-A and 21-item version of depression anxiety stress scale (DASS21) scores in CT subjects (left) and BA patients (right). Each line indicates a linear correlation for stress, anxiety, and depression scales of DASS21, respectively.

HAM-A (37) was conducted by the medical attendant who was taking care of BA patients during hospitalization. HAM-A was also conducted by the same medial attendant for a part of CT subjects who were co-workers of the medical attendant (n = 24). Consistent with the self-rating in DASS21, higher anxiety was rated for BA patients than CT subjects in HAM-A (unpaired t-test, t_38_ = 5.27, p < 0.001; **Figure 2A** and **Table 1**). Although KM patients tended to receive higher scores in this questionnaire than PP patients, this did not reach statistical significance (t_13_ = 1.91, p = 0.078; **Figure 2B**).

In CT subjects, positive correlations between HAM-A scores and each of stress (r = 0.437, p = 0.033; **Figure 2C**) and anxiety (r = 0.418, p = 0.042; **Figure 2C**), but not depression, scales in DASS21 were observed. In contrast, in BA patients, there was a positive correlation between HAM-A scores and depression (r = 0.594, p = 0.015; **Figure 2C**), but not other, scales in DASS21.

These results suggest that anxiety was higher in BA patients than that in CT subjects in the objective assessment, although correlations between self-referential and objective-rating of negative affects were not entirely consistent.

### Stress Hormone Assay and Correlation With Self-Assessments

**Figure 3:**
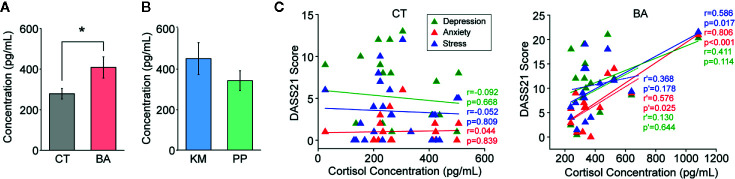
Blood cortisol concentrations and correlations with self-assessment of negative effects. **(A)** A bar graph showing cortisol concentrations in blood samples from behavioral addiction (BA) patients and control (CT) subjects. Error bars indicate s.e.m. *p < 0.05. **(B)** A graph similar to **(A)** but showing those in kleptomania (KM) and paraphilia (PP) patients. **(C)** Graphs showing correlations between cortisol concentrations and 21-item version of depression anxiety stress scale (DASS21) scores in CT subjects (left) and BA patients (right). Dashed lines with r’ and p’ indicate person’s r and p-values, respectively, excluding the outlier.

Basal blood cortisol concentrations in BA patients were significantly higher than those in CT subjects (t_38_ = 2.46, p = 0.019; **Figure 3A** and **Table 1**). Cortisol concentrations were not different between KM and PP patients (**Figure 3B**). Cortisol concentrations of BA patients were positively correlated with stress (r = 0.586, p = 0.017; **Figure 3C**) and anxiety (r = 0.806, p < 0.001; **Figure 3C**), but not depression, scales in DASS21, whereas none of these scales in DASS21 was correlated with cortisol concentrations in CT subjects (**Figure 3C**). A pattern of correlations between DASS21 and stress hormones, although significantly weaker with statistically significant correlation with only anxiety (r = 0.576, p = 0.025; **Figure 3C**) in BA patients were still maintained, even excluding an outlier, which was within the mean ± 3 standard deviation range.

These results suggest that, consistent with self-assessments, basal blood cortisol concentrations supported higher stress level in BA patients than CT subject. Moreover, correlations between self-referential negative affects and stress hormone concentrations were stronger in BA patients than CT subjects.

### Personality Traits and Associations With Negative Affects

**Figure 4:**
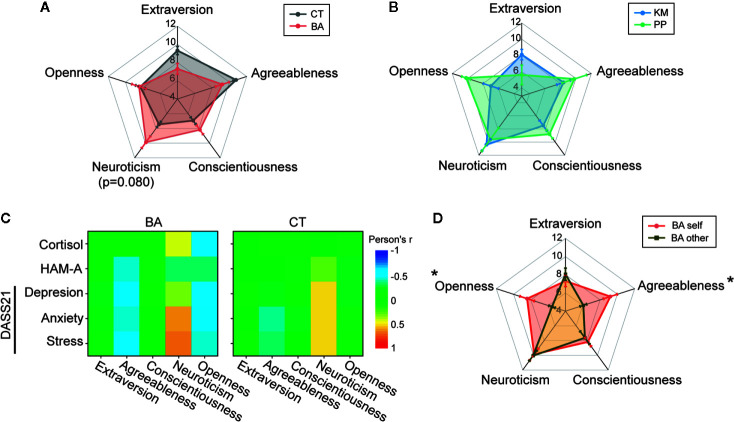
Personality traits and correlations with negative effects. **(A**, **B)** Rader charts comparing big five personality traits between behavioral addiction (BA) patients and control (CT) subjects **(A)** and between kleptomania (KM) and paraphilia (PP) patients **(B)**. Error bars indicate s.e.m. p = 0.080 in BA vs. CT at neuroticism. **(C)** A graph showing color-coded correlations (Pearson’s r) between each personality trait and negative affect assessments in BA patients and CT subjects. **(D)** A rader chart illustrating self-rating shown in **(A)** and objective rating made by others. *p < 0.05 in BA self vs. other in each trait.

In TIPI-J, BA patients self-rated their own personality traits significantly different from those of CT subjects (F_1,225_ = 0.016, p = 0.901 in group; F_4,225_ = 4.80, p = 0.001 in trait; F_4,225_ = 5.38, p < 0.001 in interaction; **Figure 4A** and **Table 1**). Post-hoc pair-wise comparisons revealed that neuroticism was marginally significantly higher in BA patients than CT subjects (p = 0.080; **Figure 4A** and **Table 1**). No statistically significant difference in any personality traits was observed between KM and PP patients (**Figure 4B**).

Correlations (Pearson’s r) of each personality trait and assessments of negative affects are illustrated in **Figure 4C**. In this analysis, positive correlations were observed between neuroticism and negative affects both in BA patients and CT subjects, with the correlations slightly stronger in BA patients than CT subjects. Moreover, negative correlations were observed between negative affects and two personality traits, agreeableness and openness, in BA patients, but not in CT subjects. However, these negative correlations seems not accurately reflect the associations between these personality traits and negative affects, given that BA patients rated themselves significantly higher agreeableness and openness than those in objective personality evaluation of BA patients given by the medical attendant (two-way ANOVA with repeated measures; F_4,75_ = 4.44, p = 0.003 in trait; F_1,75_ = 8.80, p = 0.004 in group; F_4,75_ = 4.39, p = 0.003 in interaction; post-hoc Tukey test, p = 0.015 and p = 0.035 in self- vs. objective-rating for agreeableness and openness, respectively; **Figure 4D**). Such over-rating of their own personality traits and consequent emergence of negative correlations with negative affects are reminiscent of cognitive bias in self-recognition, such as Dunning-Kruger effect (43).

Collectively, these results suggest that higher neurotic personality trait may account for higher negative affects in BA patients.

## Discussion

In this study, we have shown that negative affects, such as stress, anxiety, and depression are higher in BA patients than CT subjects, which are relatively consistent between self-referential and objective assessments. Moreover, such heightened negative affects in BA patients are associated with the stronger neurotic personality.

There are several major limitations in the current study. The most crucial limitation is a sample size. In this preliminary study, only 16 BA patients were recruited for assessments, including several association analyses. In addition, BA patients in this study were heterogeneous, divided primarily into KM and PP. Although, in all measurements, negative affects tended to be lower in PP patients than KM patients, none of them reached statistically significant difference, which is most likely due to especially the small sample size in PP patients (n = 5). Comparisons between KM and PP patients with a larger sample size will be required for the future study to further characterize whether there is difference of negative affects, depending on symptom types. Notably, we have recently shown using the genome-wide methylation analysis that DNA methylation status is different between KM and PP patients (44).

Previous studies have reported that negative affects are heightened in BA, such as pathological gambling and Internet addiction (25–27). Thus, our study extends these previous studies by demonstrating that heightened negative affects are also observed in other BA, such as KM and PP. Collectively with heightened negative affects in drug addiction (16, 17), such heightened negative affects are critical and mutual characteristics involved in addiction.

There is also a novel finding in the current study, which is different from the previous studies investigating negative effects in addiction. Assessments of negative affects in previous studies are primarily based on self-reports (25–27), whereas we examined negative affects both with self-referential and objective assessments in this study, with which correlations were found between negative affects and some of personality traits, such as openness and agreeableness, in BA patients, but not CT subjects. Moreover, the objective evaluations of personality traits for BA patients were substantially different from self-rating in openness and agreeableness. These results suggest that BA patients might overestimate some aspects of their own personality traits, which in turn is reflected as the associations with negative effects. Such associations are reminiscent of Dunning-Kruger effects, the cognitive bias on self-recognition, that poor self-recognition and low cognitive ability lead people to overestimate their own capabilities (43). Cognitive bias is under the stringent relationship with affects, and emotional states distort cognitions and decision-making (45, 46). Therefore, heightened negative effects may augment cognitive bias including the bias on self-recognition in BA. Associations of personality traits with BA have been examined in the previous studies, but the findings are inconsistent (34, 35). Such inconsistency may partly be explained by this self-recognition bias.

In our study, positive correlations were observed between self-rating of negative affects and objective assessments of anxiety with HAM-A and stress hormone with ELISA in both BA patients and CT subjects; however, these correlations were not entirely consistent. The correlations between DASS21 and HAM-A were more accurate in CT subjects than BA patients, whereas those between DASS21 and cortisol assay were better in BA patients than CT subjects. One of reasons for such limited correlations may be an involvement of cognitive bias, such as Dunning-Kruger effects (43). Another reason would be that DASS21 and HAM-A assess quite different aspects of anxiety. Thus, HAM-A assesses anxiety more heavily based on somatic aspects, such as respiratory, cardiovascular, and gastrointestinal symptoms (37), whereas DASS21 contains only some somatic assessment items (36). For cortisol assay, blood sampling was conducted different time windows of the day between BA patients (around the noon) and CT subjects (afternoon). Previous studies have shown daily fluctuations of salivary and serum cortisol concentrations, which are higher in the morning or soon after awaking, and rapidly decline before noon, and then keep slightly decreasing towards the evening (47, 48). Higher cortisol concentrations in BA patients than CT subjects may partly be due to such different timing of sampling between them; however, this does not explain better correlations with negative affects assessment with DASS21 in BA patients than CT subjects. Overall, these factors do not explain the specific pattern of correlations in BA patients and CT subjects, and therefore, the exact reason for these observations remains unclear.

In conclusion, this preliminary study with a relatively small sample size has shown heightened negative affects, such as stress, anxiety, and depression in BA patients, which is consistent with heightened negative effects in drug addiction. Such heightened negative effects are also associated with the stronger neurotic personality trait, suggesting that the neurotic personality may be one of risk factors for BA. A further study with a larger sample size of BA patients will be required to confirm whether these findings can still be retained, especially given that the results of correlations analyses reported here were decent at most.

## Data Availability Statement

The raw data supporting the conclusions of this article will be made available by the authors, without undue reservation.

## Ethics Statement

The studies involving human participants were reviewed and approved by Human Research Ethics Committee of Kyoto University Primate Research Institute, and Ethics Committee of Kyowa Hospital. The patients/participants provided their written informed consent to participate in this study.

## Author Contributions

YA, MW, and YG conceived of the research. YA, MW, TM, EI, and YG performed experiments. YA and YG analyzed the data. YA and YG wrote the manuscript. All authors contributed to the article and approved the submitted version.

## Funding

This study was supported by research grants from the Kyoto University Foundation, ISHIZUE 2020 of Kyoto University Research Development Program, Institute of Seizon and Life Sciences, and JSPS Grant-in-Aid for Challenging Exploratory Research (19K22511).

## Conflict of Interest

The authors declare that the research was conducted in the absence of any commercial or financial relationships that could be construed as a potential conflict of interest.
